# Normalization enhances brain network features that predict individual intelligence in children with epilepsy

**DOI:** 10.1371/journal.pone.0212901

**Published:** 2019-03-05

**Authors:** Michael J. Paldino, Farahnaz Golriz, Wei Zhang, Zili D. Chu

**Affiliations:** Department of Radiology, Texas Children’s Hospital, Houston, TX, United States of America; Indraprastha Institute of Information Technology, INDIA

## Abstract

**Background and purpose:**

Architecture of the cerebral network has been shown to associate with IQ in children with epilepsy. However, subject-level prediction on this basis, a crucial step toward harnessing network analyses for the benefit of children with epilepsy, has yet to be achieved. We compared two network normalization strategies in terms of their ability to optimize subject-level inferences on the relationship between brain network architecture and brain function.

**Materials and methods:**

Patients with epilepsy and resting state fMRI were retrospectively identified. Brain network nodes were defined by anatomic parcellation, first in patient space (nodes defined for each patient) and again in template space (same nodes for all patients). Whole-brain weighted graphs were constructed according to pair-wise correlation of BOLD-signal time courses between nodes. The following metrics were then calculated: clustering coefficient, transitivity, modularity, path length, and global efficiency. Metrics computed on graphs in patient space were normalized to the same metric computed on a random network of identical size. A machine learning algorithm was used to predict patient IQ given access to only the network metrics.

**Results:**

Twenty-seven patients (8–18 years) comprised the final study group. All brain networks demonstrated expected small world properties. Accounting for intrinsic population heterogeneity had a significant effect on prediction accuracy. Specifically, transformation of all patients into a common standard space as well as normalization of metrics to those computed on a random network both substantially outperformed the use of non-normalized metrics.

**Conclusion:**

Normalization contributed significantly to accurate subject-level prediction of cognitive function in children with epilepsy. These findings support the potential for quantitative network approaches to contribute clinically meaningful information in children with neurological disorders.

## Introduction

Pediatric epilepsy is a prototypical disorder of network dysfunction: even in the setting of a highly localized structural lesion, children with epilepsy demonstrate widespread alterations in cerebral cortical networks[1,2]. Whether established by genetic/developmental processes or by activity-dependent reorganization, and there is evidence to support a role for each, global network dysconnectivity undermines the brain’s capacity to support normal neuro-cognitive development[3–5]. Furthermore, the negative effects of epilepsy on intellectual function seem to be exaggerated in children, which may reflect the fact that developmental physiology is primed for cerebral growth and network reorganization[6]. Regardless of origin, the impact of network dysfunction can be seen in the range and severity of cognitive failings exhibited by these children, often far beyond what would be expected based on the location and extent of their structural abnormalities[7]. The ability to understand and predict the impact of global network dysconnectivity in an individual child with epilepsy would be of great value to the care of these patients. Current understanding points to the emergence of cognitive function from complex interactions occurring across large-scale brain networks that support both segregation into, as well as integration across, subspecialized systems[8]. Non-invasive methodologies that capture the organization of the brain as a network of interacting elements, therefore, represent an appealing approach by which to study neurologic dysfunction in children with epilepsy.

Resting-state functional MRI, which measures the blood oxygen level-dependent (BOLD) signal over time, is one method by which connectivity within a neural network can be measured[9]. Its acquisition in the MR scanner does not require cooperation with task paradigms and can even be acquired under sedation[10]; it is therefore of particular value to the young or developmentally impaired. Elements of the cerebral cortex that interact to support a given function continue to exhibit similar spontaneous BOLD fluctuations at rest[11]. Functional connectivity, defined as the magnitude of this similarity, can therefore be used to create a comprehensive map of connections in the brain[12]. Within this framework, the brain is represented as a collection of “nodes”, or anatomical elements in the network, and the connection between each pair of nodes as an “edge”. Although the field capitalizes on diverse techniques, one prominent approach leverages graph theory to characterize global topological features of the cerebral network[12]. An array of graph theory metrics has been described, each of which has the potential to capture specific topological features of the network[13]. In general terms, though, these graph metrics measure in some way the degree to which the network supports either integration (efficient exchange of information) across, or segregation (the network substrate for functional sub-specialization) within, the brain. Integration and segregation have been found to be critical features of brain function in healthy populations of adults and children[14–16] as well as in children with focal epilepsy[17]. Network features have also been shown to capture the pathophysiologic impact of epilepsy on the cerebral network[18].

A prodigious body of work has leveraged resting state constructs to characterize features of patient groups that deviate from groups of normal subjects[19–21]. Yet in the clinical realm, it is more often the variation *within* a patient group–for example, disease severity, stage, prognosis—that is most relevant to patient care. Recent work has demonstrated the potential of machine learning to translate continuous quantitative imaging data into meaningful subject-level markers[18,22–24]. One major obstacle to realizing the full promise of these techniques, however, is the inter-individual variation observed in all populations, even those comprised of normal adults[19]. This obstacle is exaggerated in pediatric cohorts whose individual trajectories of brain development add to the otherwise expected variation. Variation in network scale in particular has been shown to be an important potential confound—global properties of a given brain vary considerably with the number of nodes arbitrarily defined therein. Growth of the brain over the course of normal development therefore represents a significant obstacle for those that aim to apply these techniques in children[25,26]. Methods that capture network organization in a way that generalizes across development would be of great value in this regard.

Developmental variation in brain networks is most commonly addressed by transforming the imaging data from all patients into a common three-dimensional space by registration to a standard template[27]. In this *network registration* strategy, every patient’s network is theoretically constructed using the same nodes at identical locations in template space. However, transformation makes assumptions regarding similarity in shape and folding pattern that may not be valid, particularly in a pediatric cohort[28]. It therefore has the potential to remove or obscure some of the inter-subject variation that is actually meaningful to individual brain function. Recent work has demonstrated potential benefit in computing network measures in each patient’s native space[28]. To account for differences in scale, the output network metrics can then be normalized to the same metric computed on a null or random network model of identical size[29]. Although this *metric normalization* strategy avoids errors in misregistration related to individual differences in brain morphology, it remains largely untested. The goal of this study was to compare these two fundamental strategies in terms of their ability to optimize subject-level inferences on the relationship between brain network architecture and brain function.

## Material and methods

### Study population

This HIPAA-compliant, retrospective study was approved by the Baylor College of Medicine institutional review board. Written informed consent was waived for this study using imaging data already in existence in the medical record. Consecutive patients were identified from the medical record according to the following inclusion criteria: 1. Pediatric age group (less than 21 years of age); 2. a clinical diagnosis of focal epilepsy[30] established by a pediatric epileptologist based on clinical history and semiology; 3. A 3 Tesla MRI examination of the brain, including a resting state fMRI sequence, performed after a major scanner upgrade (multi-transmit/multi-receive) in April, 2013; 4. Full-scale intelligence quotient measured according to an age-appropriate version of the Wechsler Intelligence Test administered by a pediatric neuropsychologist within 3 months of the MRI. Refinements to the above-defined population were planned based on the following exclusions: 1. prior brain surgery. Intelligence tests were performed by a single pediatric neuropsychologist with more than 25 years experience in pediatric epilepsy.

### Magnetic resonance imaging

Imaging was performed on a 3 Tesla magnet (Philips, Achieva, Andover, Massachusetts) using a 32-channel phased array head coil. The following sequences were performed for each patient: Structural Imaging: T1-weighted, three-dimensional volume acquisition fast field echo (TR/TE: 7.2/2.9 ms, flip angle: 7 degrees, TI: 1100 ms, voxel size: 0.9 x 0.9 x 0.9 mm^3^); Functional Imaging: Single-shot echo planar blood oxygenation level dependent images (TR/TE: 2000/30 ms, flip angle: 80^o^, voxel size: 3 x 3 x 3.75 mm^3^). Functional images were acquired in the resting state for 10 minutes (300 volumes) for each patient. Patients were instructed to lie quietly in the scanner with their eyes closed. All images were visually inspected for artifacts, including susceptibility and subject motion.

### Image processing and analysis

The common processing pipeline was implemented using MATLAB scripts (version 7.13, MathWorks, Inc) in which adapter functions were embedded to execute FreeSurfer reconstruction (version 5.3.0; http://surfer.nmr.mgh.harvard.edu) as well as several tools from the FMRIB Software Library (FSL)[31]. Details regarding this pathway have been previously described[18,23]. An overview is provided here:

#### Network node definition

Nodes in the network were defined according to parcellation of whole-brain gray matter. First, FreeSurfer reconstruction of cerebral cortical surfaces was performed on the T1 structural image according to the Destrieux Atlas[32]. FreeSurfer was selected for this task as surface based registrations that take into account sulcal and gyral anatomy are likely to improve subject to subject comparison and thereby minimize inter-subject variability[19]. This processing stream included motion correction, skull stripping, segmentation of white matter and gray matter structures, surface deformation following intensity gradients which optimally place the gray matter/white matter and gray matter/CSF borders, and parcellation of the gray matter and white matter boundary[33,34]. Pial and gray white surfaces were visually inspected using the Freeview software for accurate placement. Next, a self-developed MATLAB program was applied to the FreeSurfer output to further subdivide the 74 standard gray matter parcels (per hemisphere) until they reached the desired size. This step utilized the FreeSurfer output white surface, which is a 3D triangulated surface mesh placed at the boundary of the gray and white matter of the brain, and the standard gray matter (cortical) parcels generated according to the Destrieux atlas[32]. At each iteration, existing cortical parcels were evaluated according to their surface area on the white surface mesh. For parcels greater in surface area than the predetermined size threshold (see below for details regarding size threshold definition), that parcel was divided into two smaller parcels of equal size as follows: 1. the distance (along the white surface) between all vertices within a parcel were measured. 2. the largest distance between vertices within a parcel was defined as vector n; 3. the original parcel was divided into two new cortical parcels along a line perpendicular to n. This process continued until all parcels were smaller than the predetermined size threshold. Each surface parcel was then converted into a volume mask of gray matter (cortex) at that region to form a node on the network. This kind of random (not conforming to any known anatomic boundary) subdivision of standard cortical parcels in order to construct larger network sizes (larger number of nodes) is standard practice going back to the origin of the field[12,25]. It has been shown that, although the network properties are affected by the network size, nodes defined in this manner retain their network properties[25,35].

#### Network edge definition

The first 5 volumes in each resting state fMRI data were removed to allow magnetization to reach equilibrium. Preprocessing and independent component analysis (ICA) of the functional data sets was performed using FSL MELODIC[31], consisting of motion correction, interleaved slice timing correction, brain extraction, spatial smoothing with a Gaussian kernel full width at half maximum of 5 mm, and high pass temporal filtering equivalent to 100 seconds (0.01 Hz). Noise related to motion and other physiologic nuisance was addressed according to an ICA technique[36]. All non-signal components were identified manually by an expert operator. FSL’s FLIRT was then used to align the functional image volumes for each patient to that individual’s structural T1 dataset using linear registration. The inverse transformation matrix was calculated in this step and subsequently used to transform all masks from structural to functional space. Mean BOLD-signal time series were then extracted on the previously defined nodes. The strength of an edge between two nodes was defined as the absolute value of the Pearson correlation coefficient between their time series.

#### Generation of weighted graphs

Weighted, undirected graphs were constructed for each patient consisting of the pair-wise correlation between BOLD signal time series over all network nodes. Non-significant correlations were excluded based on Bonferroni adjusted (to account for multiple comparisons) *p-*values thresholded at 0.05.

#### Network metric calculation

For each weighted, undirected connection matrix, the following graph theoretical metrics were calculated using The Brain Connectivity Toolbox (http://www.brain-connectivity-toolbox.net): clustering coefficient, transitivity, modularity, characteristic path length, and global efficiency. A brief description for each metric is provided in Table 1. These metrics were selected as they each capture the degree to which the network supports either integration across, or functional sub-specialization within, the brain[13].

**Table 1:** Graph theoretical metrics of global network architecture.

Metric	Description
Clustering Coefficient	The fraction of a given node’s neighbors that are also neighbors of each other. Reflects segregation/subspecialization in a network
Transitivity	The fraction of node threesomes in the network that form a completely connected triangle; a variant of clustering coefficient. Reflects segregation/functional subspecialization in a network
Modularity	The degree to which nodes tend to form relatively independent modules or subnetworks. Reflects segregation/subspecialization within a network
Characteristic Path Length	The number of network edges required to traverse the distance between two nodes. Reflects the ease of information transfer across the network
Global Efficiency	The inverse of the shortest path lengths between two nodes averaged over the network. Reflects integration in a network

#### Normalization

Within the confines of the above common processing pipeline, two different strategies were applied to account for expected variations of scale inherent to a clinical pediatric cohort:

Network registration:In this condition, prior to entering the common processing pipeline, structural imaging data for each patient were aligned to a standard template (Montreal Neurological Institute (MNI) 152) using non-linear registration[37–39]. Node definition was performed on the template itself. Therefore, every patient’s network was constructed using the same nodes in identical locations in the standard template space.

In detail, we used FSL FLIRT to accomplish linear boundary-based registration between each patient’s functional and structural images; we also used nonlinear 12 degree-of-freedom registration to transform each patient’s structural images into the standard template (MNI152). A single node parcellation for all patients was performed. Nodes were generated using the strategy above (network node definition) using one patient selected from our pediatric cohort based on the best registration to MNI space. This strategy was selected as it has been shown to reduce potential confounds related to registration in a pediatric cohort[39].

Metric normalization:In this condition, by contrast, network nodes were defined individually for each patient in their native space. After metric generation via the common processing pipeline, each network metric was then normalized to the same metric computed on a random network of identical size and conserved degree, strength, and weight distribution[29].

Raw metrics:In this condition, network measures were not normalized in any fashion. Nodes were defined individually for each patient in their native space, with no metric normalization.

#### Network size

The optimal number of network nodes for the purpose of patient-level prediction is unknown[25]. Therefore, networks were constructed at multiple sizes. This was accomplished by defining three different size thresholds during network node definition: 600 mm^2^, 350-mm^2^, and 150 mm^2^. This step resulted in networks of approximately 420, 705, and 1620 nodes for each patient. These sizes were selected in order to cover the span of network sizes commonly seen in the literature.

### Statistical analyses

All statistical analyses were performed using R Language, version 3.0.2 (R Foundation for Statistical Computing, Vienna, Austria).

In the primary analysis, a random forest machine learning algorithm was used to predict each patient’s full scale IQ on the basis of the five global network metrics. In this step, the algorithm was given access solely to the network metrics and no other variables. This machine learning method tests the predictive capacity of a “learned” statistical model on a subset of the cohort omitted during training. In other words, the ability of the model to predict IQ in each individual was tested in a previously unseen subset of patients. Details regarding this particular technique have been previously described[40]. In brief, Random Forests are an ensemble learning method that operates by constructing a multitude of decision trees during training. The forest is then used to make predictions that reflect the average output from the individual decision trees. During training, approximately one third of the cohort is omitted at random from the training set—this portion of the dataset is considered “out-of-bag”. The IQ of each individual held out of bag is then predicted based on the “learned” model. Prediction accuracy for each condition was then compared over the cohort using fractional variation explained[41].

As a control, an alternative predictive model was developed with only potential confounders, including physiologic (age, gender, total cortical volume, and the number of network nodes) and nuisance variables (rotational and translational motion during MRI). To be specific, network metrics were not included in the control model. The control model was then tested for its ability to predict full-scale IQ using an otherwise identical Random Forest algorithm.

Linear regression was used to assess potential relationships between continuous variables, including age, cortical volume, and full scale IQ (alpha: 0.05).

## Results

### Patients

Thirty-six patients met inclusion criteria. Ten were excluded on the basis of prior brain surgery. Hence, twenty-six patients with focal epilepsy (age range: 8–18 years) comprised the final study group. Five patients had structurally normal brains at anatomic MRI; twenty-one had structural abnormalities. Patient characteristics of the cohort are provided in Table 2. All patients were imaged as part of the evaluation for surgical management of their epilepsy. The Wechsler Intelligence Scales for Children (WISC-IV) was successfully administered in all patients[42]; full scale intelligence quotient in the cohort ranged from 52 to 129. As expected, cortical volume was significantly related to age (*p*: 0.018). However, neither age (*p*: 0.45) nor cortical volume (*p*: 0.34) were significantly related to IQ.

**Table 2:** Characteristics of the patient cohort.

Patient Characteristics		
Sample Size	26 patients	
Gender	14 males; 12 females	
Age	Mean (SD): 13.9 (3.0) years	
Full Scale IQ	Mean (SD): 89 (17)	
Age of onset	Mean (SD): 5.3 (4.2) years	
Duration of Epilepsy (at time of MRI)	Mean (SD): 8.6 (5.3) years	
Medications (number) at time of MRI	Median (range): 3 (1–8)	
Anesthesia during MRI	9/26 patients	
MR Structural Lesions	Focal cortical dysplasia	9
	Mesial temporal sclerosis	5
	Normal MRI	5
	Low-grade tumor	4
	Tuberous sclerosis Complex	3

### Network construction

In standard space, each patient’s network consisted of 1620, 705, and 420 nodes for the small (150 mm^2^), intermediate (350mm^2^) and large (600 mm^2^) node thresholds respectively. Network sizes constructed in patient space for the three node size thresholds are summarized in Table 3. As described above, the number of nodes defined in patient space relates to the volume of the cortex in each child; in turn, this number is correlated with the age of the child. Mean network sizes for the two conditions did not differ significantly at any of the node size thresholds. For networks constructed in patient space, there was no significant association between the number of nodes and IQ (*p*: 0.37). All brain networks demonstrated small world properties, characterized by a tendency to form communities (clustering coefficient greater than that of a random graph) while at the same time maintaining efficiency (path length approximating that of a random graph).

**Table 3:** Summary of network sizes when constructed in standard space and patient space.

Node Size	Network Nodes	
	Standard Space	Patient Space
600 mm^2^	420	420 [35]
350 mm^2^	705	705 [65]
150 mm^2^	1620	1620 [144]

For standard space, all subjects have the same number of network nodes for a given node size threshold. For patient space, the mean [standard deviation] nodes per network are provided for the cohort.

### Network metrics and intelligence

**Fig 1:**
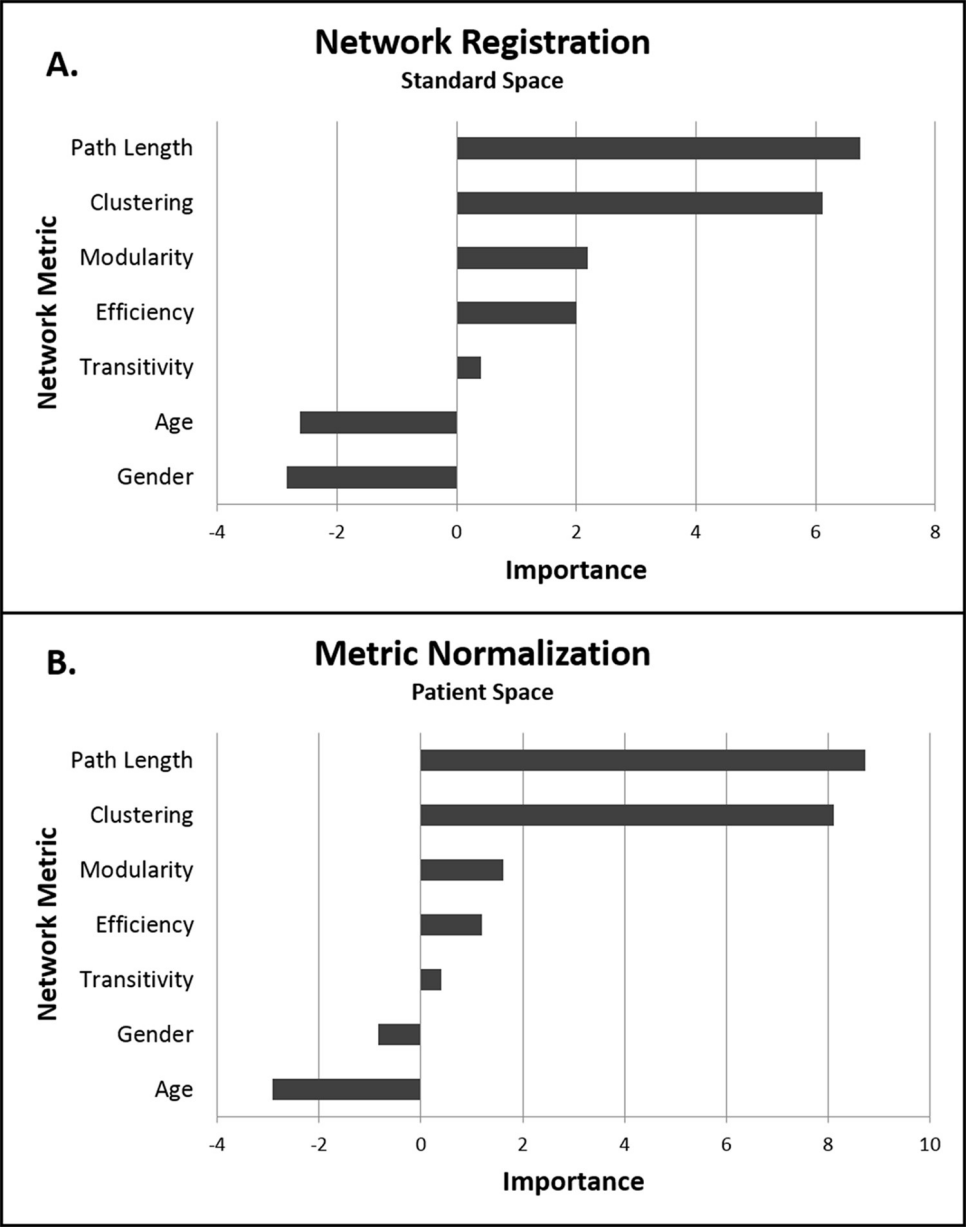
Importance of global metrics of network architecture to intelligence quotient (IQ) prediction. Metrics were computed in (a) standard space or (b) in patient space with normalization of output metrics to a random network of the same size. The independent contribution of each metric was estimated as the error of the learning algorithm’s IQ prediction compared to the error which results when that metric is negated. The most negative value of importance defines the limit of noise. Hence, variables with importance greater in magnitude than the most negative variable are significant.

Accuracy of the machine learning algorithm for patient IQ prediction is presented in Table 4. Prediction accuracy consistently increased with the number of nodes in the constructed network. Accounting for network scale had a significant effect on prediction accuracy at all node sizes. Specifically, both normalization strategies significantly outperformed the use of raw metrics (derived from networks constructed in patient space without normalization to metrics computed on a random network). Metric normalization in patient space demonstrated higher accuracy than network registration into standard space for all but the largest nodes (Table 4). The choice of normalization strategy did not alter the specific network features most important to full scale IQ; path length and clustering coefficient were the dominant metrics contributing to IQ prediction in both conditions (Fig 1). The control model based on covariates only (age, gender, total cortical volume, number of network nodes and patient motion during image acquisition) was a poor predictor of patient IQ (fractional variation explained [95%CL]: 0 [-0.03, 0.03]). Metrics computed on random networks (ie. metrics used for normalization) were also poor predictors of full–scale IQ, consistent with the idea that metric normalization did not introduce potentially useful variation into the model.

**Table 4:** Fractional variation explained [95% confidence limits] of the machine learning algorithm for predicting patient IQ based on network metrics.

Node Size	None	Metric Normalization	Standard Space
600 mm^2^	0.03 [0.01, 0.05]	0.17 [0.14, 0.20]	0.14 [0.12, 0.18]
350 mm^2^	0.05 [0.02, 0.08]	0.31 [0.28, 0.34]	0.17 [0.14, 0.20]
150 mm^2^	0.08 [0.05, 0.11]	0.34 [0.31, 0.37]	0.20 [0.17, 0.23]

## Discussion

We evaluated two strategies that aim to address the inter-individual variation in brain networks inherent to a clinical pediatric cohort. We specifically assessed the impact of these strategies on output metrics of global brain architecture in terms of their capacity to support the prediction of global intelligence in children with focal epilepsy. We report the following primary findings: 1. Normalization by either strategy significantly improved subject-level prediction of global intelligence; 2. Metric normalization in patient space outperformed the use of network registration into standard space under most conditions; and 3. Prediction improved across all conditions with increasing nodes in the network.

Higher order functions of the brain emerge from parallel processing within sub-specialized, but distributed, functional systems. A complex neural network formed by approximately 10^10^ neurons forms the structural substrate for efficient communication across the cerebrum. Within this network, segregation into relatively independent local neighborhoods provides an architectural framework for functional sub-specialization. Yet a complete range of function only emerges from efficient integration of these sub-specialized neighborhoods across the entire brain. Small world organization–characterized by both high clustering coefficients and short path lengths—is an effective means by which both functional sub-specialization and integration can be concomitantly supported by the same network. It has been suggested that the efficiency derived from small world organization contributes to the biologic underpinnings of cognitive function in the human brain[15,43,44]. Only recently, however, have we begun to explore the primary role that dysfunction of this network plays in the pathogenesis of human disease. At the leading edge of such initiatives are mathematical approaches to quantitative imaging that model the human brain specifically as a network of discrete, interacting elements[12]. Optimal methods by which to create such constructs, however, remain the subject of debate. These inconsistencies reflect, at least in part, the multiplicity of anatomic and functional scales across which the human brain is organized[45]. At one extreme, encoding each neuron as a node in the network represents an intuitive schema by which to depict the true organization of the cerebral network. Techniques that reliably probe the brain at this scale, however, are not yet widely available. At the macroscopic scale, by contrast, large scale brain networks are accessible to systematic study through noninvasive MR technologies, including diffusion weighted imaging and BOLD acquisitions. At this scale, however, there are no objective criteria by which to establish boundaries between elements in the network[25]. As a consequence, investigators have parcellated the cortex according to an array of definitions, a strategy which has led to a relative lack of comparability across studies[19]. The gravity of this problem was underscored by Zalesky et al. who demonstrated large (up to 95%) variations in global network properties solely on the basis of network scale[25]. Within any given study, this problem is substantially mitigated by internal consistency; the same node definition results in a similar number of nodes across subjects. In a pediatric cohort, however, the diversity of developmental stages within the study population contributes to a range of network sizes even for the same node definition. Prediction using machine learning would likely benefit from strategies that account for this diversity such that the entire population can be considered by a cohesive approach. We observed that two such strategies–network registration by transformation of all patients into a common template space and metric normalization to the same output metric computed on a random network of identical size–significantly enhanced prediction of a child’s IQ based on the global network properties of his/her brain. Overall, the findings support the potential for normalization to improve subject-level inferences from resting state networks. These results are consistent with the small but growing body of evidence that single sessions of resting state fMRI contain sufficient information to make predictions about individuals[46], including those with focal epilepsy[22].

We also observed that normalization of metrics computed in each patient’s native space supported more accurate prediction than network registration into standard space. Under ideal circumstances, the standard space solution considers the same nodes in the same location in every patient. However, the variation in cortical shape and folding patterns inherent to a pediatric population poses a challenge to accurate registration of individual brains to the standard template[19]. Even small errors in registration result in variable placement of nodes across the cohort, thereby altering the global properties of individual networks in random fashion[35]. Given that they contribute variation that is irrelevant to intellect, such errors could explain the reduced accuracy of the learning algorithm. Alternatively, it is possible that brain shape and folding might actually be meaningful to patient intelligence[47]. Removal of this variation during transformation to a standard template, therefore, could also contribute negatively to prediction. A final possible explanation lies in the potential diagnostic value imparted specifically by normalizing metrics to a random network. By comparing to a network with no consistent organization, metric normalization emphasizes the degree to which a given network tends to form local communities within a small world framework. Given the suggestion that small world properties underpin, at least in part, the development of cognitive function, it would not be surprising to find added value in methods that highlight such features[15,43,44]. It is important to note, these potential explanations need not act in isolation; all three or any combination thereof could have contributed to the findings observed in this study. Regardless of origin, our results are consistent with previous work that has demonstrated the impact of node definition on global metrics derived from brain networks[25,28] and, further, has suggested the potential superiority of parcellation in the patients’ native space[28].

The above discussion has centered on the variation in network scale inherent to a pediatric cohort for the same node size. However, the optimal size of network nodes is also yet to be established[25]. During network construction, large nodes potentially include adjacent but functionally distinct cortical regions into a single region of interest. The averaged BOLD time course from a large node, therefore, may not accurately reflect the actual time-course of any of the functional regions contained therein[48]. Measuring pair-wise correlations between such time courses would then be handicapped with respect to its ability to capture the brain’s true interactions. By contrast, small nodes are accompanied by lower signal-to-noise ratios, all other parameters being equal; they also add to the computational burden of network analyses. At some point, parcellation schemes may become noise-limited. Hence, optimal parcellation should balance these competing concerns in such a way that global metrics most closely reflect true brain network topology. We observed that IQ prediction improved across all conditions with increasing nodes in the network. This finding is consistent with the idea that, over the range of node sizes commonly reported in the literature, the benefits of node homogeneity may outweigh the competing costs to SNR. It remains to be seen, however, whether further increases in network scale would continue to strengthen the relationship between brain network architecture and brain function.

Consistent with previous work, we observed that full scale IQ was directly related to clustering coefficient (segregation) but inversely related to path length (integration)[17,49]. The association of short path lengths with lower cognitive function has been shown to be statistically mediated by seizure duration, suggesting that ongoing seizures are associated with rewiring of the cerebral network[18]. These findings reinforce the idea that network metrics in epileptic brains may not have the same physiologic meaning as in normal subjects. Synaptic efficacy, according to Hebbian theory on neural plasticity, arises from repeated and persistent stimulation[50]. In this manner, connections contributing to useful and efficient sub-networks are strengthened over time, while those associated with less functional/inefficient networks are pruned[51]. In the setting of epilepsy, however, synapses are strengthened along pathways related to seizure propagation, essentially hijacking Hebbian processes[52]. Connectivity in this setting is potentiated without regard to network function, resulting in aberrant and potentially maladaptive pathways[53,54].

This study has several limitations. First, it was performed in a selected cohort of pediatric patients with focal epilepsy. Generalization of these results to other patient groups would require further study. Analogously, we studied the impact of these strategies specifically on global metrics derived from a network graph theory approach to resting state fMRI. Optimal methods for other types of resting state data analysis, including the study of intrinsic resting state functional networks, will not necessarily parallel those presented here. Third, all imaging was performed on the same MR scanner with the same phased array coil according to the same resting state fMRI sequence. Further study regarding the utility of these metrics across a wider range of MR hardware would be of great practical value to widespread implementation. Finally, the goal of this study was to promote the development of methods that remove or mitigate the effects of normal variation, thereby highlighting those features most relevant to individual brain function. However, the boundary between variation that is “normal” and that which is clinically important may not always be clear. Along similar lines, variation that is important in one context may be irrelevant under other circumstances. It is therefore possible that alterations of the data inherent to these and similar techniques could, albeit unintentionally, remove variation that is clinically important under some circumstances. Ultimately, the success of machine learning in pediatric neuroimaging will rely on the development of patient databases large enough to allow the algorithm to learn on its own which variation is relevant to each application.

## Conclusion

In conclusion, normalization contributed significantly to the prediction of individual intelligence in a cohort of children with focal epilepsy. Both of the tested normalization strategies significantly augmented prediction by the learning algorithm. However, under most conditions, normalization of metrics computed in patient space outperformed transformation of all patients into a standard space. These findings support the potential for network science to provide clinically meaningful markers of brain function in children with epilepsy.
